# Long-term safety and efficacy of tocilizumab, an anti-IL-6 receptor monoclonal antibody, in monotherapy, in patients with rheumatoid arthritis (the STREAM study): evidence of safety and efficacy in a 5-year extension study

**DOI:** 10.1136/ard.2008.092866

**Published:** 2008-11-17

**Authors:** N Nishimoto, N Miyasaka, K Yamamoto, S Kawai, T Takeuchi, J Azuma

**Affiliations:** 1 Osaka University, Osaka, Japan; 2 Tokyo Medical and Dental University, Tokyo, Japan; 3 University of Tokyo, Tokyo, Japan; 4 Toho University Omori Medical Center, Tokyo, Japan; 5 Saitama Medical Center/School, Saitama, Japan

## Abstract

**Objectives:**

To evaluate the safety and efficacy of 5-year, long-term tocilizumab monotherapy for patients with rheumatoid arthritis.

**Methods:**

In an open-label, long-term extension trial following an initial 3-month randomised phase II trial, 143 of the 163 patients who participated in the initial blinded study received tocilizumab monotherapy (8 mg/kg) every 4 weeks. Concomitant therapy with non-steroidal anti-inflammatory drugs and/or oral prednisolone (10 mg daily maximum) was permitted. All patients were evaluated with American College of Rheumatology (ACR) improvement criteria, disease activity score (DAS) in 28 joints, and the European League Against Rheumatism response, as well as for safety issues.

**Results:**

143 patients were enrolled in the open-label, long-term extension trial and 94 (66%) patients had completed 5 years as of March 2007. 32 patients (22%) withdrew from the study due to adverse events and one patient (0.7%) due to unsatisfactory response. 14 patients withdrew because of the patient’s request or other reasons. The serious adverse event rate was 27.5 events per 100 patient-years, with 5.7 serious infections per 100 patient-years, based on a total tocilizumab exposure of 612 patient-years. Of the 88 patients receiving corticosteroids at baseline, 78 (88.6%) were able to decrease their corticosteroid dose and 28 (31.8%) discontinued corticosteroids. At 5 years, 79/94 (84.0%), 65/94 (69.1%) and 41/94 (43.6%) of the patients achieved ACR20, ACR50, and ACR70 improvement criteria, respectively. Remission defined as DAS28 less than 2.6 was achieved in 52/94 (55.3%) of the patients.

**Conclusion:**

In this 5-year extension study, tocilizumab demonstrated sustained long-term efficacy and a generally good safety profile.

Rheumatoid arthritis (RA) is a chronic inflammatory disease characterised by persistent synovitis and progressive joint damage.^1^ Although the causes of RA are not fully understood, constitutive overproduction of IL-6, a multifunctional cytokine that regulates the immune response, inflammatory reaction and bone metabolism, is thought to play a major pathological role in RA.^2^

Tocilizumab is a humanised anti-human IL-6 receptor monoclonal antibody,^2^ which has been demonstrated to improve the signs and symptoms of RA^3^ ^4^ ^5^ ^6^ ^7^ ^8^ ^9^ and prevent radiographic progression^10^ in previous clinical trials. Those controlled trials provided evidence for a rapid reduction in disease activity in response to tocilizumab in patients with active RA as measured by American College of Rheumatology (ACR) responses, disease activity scores (DAS) and a modified health assessment questionnaire (MHAQ).^5^ ^6^ ^7^ ^8^ ^9^ The efficacy was dose related and 8 mg/kg tocilizumab provided a marked clinical benefit. The success in the treatment of patients with RA using tocilizumab confirmed that IL-6 plays an important pathological role in RA, and further studies were therefore required to determine the long-term safety and efficacy of tocilizumab treatment. We report here the safety and efficacy of tocilizumab in a 5-year long-term extension study.

## Methods

### Patients

This study was registered with http://www.clinicaltrials.gov (NCT00144651). The study protocol was approved by the Ministry of Health, Labor and Welfare of Japan and by the ethical committee of each institute, and patients gave their written informed consent.

The eligibility criteria and the study design of the initial 12-week, randomised, double-blind, placebo controlled study have been reported previously.^5^ Briefly, eligible patients were 20 years of age or older and fulfilled the 1987 criteria for RA of the American Rheumatism Association^11^ with a disease history of longer than 6 months. All subjects had been insufficient responders to treatment with at least one disease-modifying antirheumatic drug (DMARD) or immunosuppressant. Patients had active disease at the time of enrollment into the initial controlled trial, as defined by the presence of six or more swollen joints, six or more tender joints and one of the following two criteria: a Westergren erythrocyte sedimentation rate (ESR) of at least 30 mm/h or a C-reactive protein (CRP) level of more than 1.0 mg/dl. Patients receiving prednisolone (10 mg daily maximum) and/or non-steroidal anti-inflammatory drugs (NSAID) were eligible if the dose had not increased during the washout period of 1 month. Doses of both medications remained stable during the blinded study period of 12 weeks. Patients who had received tocilizumab or placebo twice or more were given the opportunity to receive tocilizumab in this open-label extension trial.

In the extension study, the use of prednisolone (10 mg daily maximum) and one NSAID was permitted. Sexually active premenopausal women were required to have a negative urine pregnancy test at entry and to use effective contraception during the study period.

### Treatment

Patients were randomly assigned to receive either placebo, or 4 or 8 mg/kg body weight of tocilizumab every 4 weeks in the initial blinded 12-week trial. In the first 12 weeks of the open-label extension study, patients received 8 mg/kg tocilizumab every 4 weeks and thereafter dose reduction and treatment interval changes (minimum 2 weeks) were allowed.

### Efficacy assessments

Disease activity was assessed at baseline and at every visit during the initial blinded trial and the first 12 weeks of the extension study, and thereafter every 3 months. All patients were evaluated with ACR improvement criteria, DAS28 and the European League Against Rheumatism response. The DAS28 was calculated using the ESR. Clinical assessments included the following: complete counts of swollen and tender joints (49 joints evaluated; cervical spine and hips evaluated only for tenderness); physician’s and patient’s global assessment of disease status, on a visual-analogue scale from 0 (asymptomatic) to 100 (severe symptoms); patient’s assessment of pain on a scale from 0 (no pain) to 100 (severe pain); functional disability measured with a MHAQ; ESR and CRP levels.^12^ Treatment time was calculated beginning with the first infusion of tocilizumab, excluding the time receiving placebo.

### Safety assessments

Safety was assessed for all patients who received at least one dose of tocilizumab in the extension study. Serious adverse events (SAE) were defined as events that were fatal or life-threatening, leading to permanent or significant disability or incapacity, a congenital anomaly or birth defect, or requiring prolonged inpatient hospitalisation. Adverse events were classified using the Medical Dictionary for Drug Regulatory Affairs (MedRA version 8.0).

### Statistical analysis

Patients who had remained in the study and had completed visit reports were analysed. No imputation was used for missing data. A paired t test was employed to detect statistically significant differences in disease activity and functional outcomes from baseline. Statistical analyses were performed with SAS version 8.2 TS2M0. The continuation rate, defined as the cumulative percentage of patients still receiving medication, was analysed using the Kaplan–Meier method. Analysis of adverse events was performed with the person-year method.

## Results

### Characteristics of the patients

**Figure 1:**
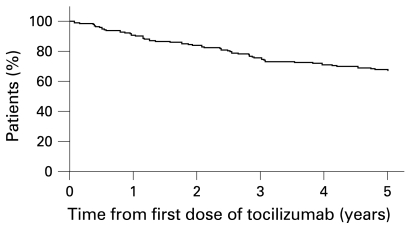
Kaplan–Meier estimate of the probability of the patients remaining on study. Treatment time was calculated beginning with the first infusion of tocilizumab at any dose, excluding the time receiving placebo.

A total of 143 patients was enrolled in the open-label, long-term extension trial; 108 patients (76%) had completed 3 years and 94 patients (66%) had completed 5 years, as of March 2007 (fig 1). The median duration of treatment with tocilizumab was 66.7 months (range 0.95–73.2).

Thirty-two patients (22%) withdrew due to adverse events. Only one patient (0.7%) withdrew due to unsatisfactory response. Other reasons for withdrawals were as follows: eight for patient’s personal requests; one for the emergence of anti-tocilizumab antibodies and five for other reasons.

The baseline demographic and clinical data are summarised in table 1. The patients’ mean age was 54 years and the mean disease duration was 9.9 years. Patients had very active disease at baseline, in terms of the increased number of tender and swollen joint counts and elevated ESR of 68.7 mm/h and CRP levels of 4.7 mg/dl. Furthermore, the baseline DAS28 was 6.7.

**Table 1:** Demographics and baseline clinical characteristics of patients with RA who received tocilizumab at any time during the blinded period or open-label extension of the tocilizumab study

	Tocilizumab (n = 143)
Demographics	
Age, years (SD)	54.3 (11.1)
No of men/no of women	34/109
Clinical characteristics	
RA duration, years (SD)	9.9 (8.4)
No of failed DMARD, mean (range)	4.5 (1–11)
Functional class,* I/II/III/IV	10/93/40/0
RA stage,* I/II/III/IV	3/34/56/50
Tender joint count, 0–49 scale (SD)	20.3 (10.3)
Swollen joint count, 0–46 scale (SD)	14.5 (8.7)
ESR, mm/h (SD)	68.7 (29.9)
CRP, mg/dl (SD)	4.7 (3.3)
DAS28 (SD)	6.7 (1.0)

Values are mean (SD) unless stated otherwise. The data were calculated from the baseline of the double-blind trial (4 mg/kg group, 8 mg/kg group) and from the extension trial (placebo group). *Rheumatoid arthritis (RA) functional status determined by American College of Rheumatology criteria. RA stage determined by Steinbrocker’s criteria. CRP, C-reactive protein; DAS28, disease activity score in 28 joints; DMARD, disease-modifying antirheumatic drugs; ESR, erythrocyte sedimentation rate.

### Safety

A total of 148 SAE was reported in 77 patients (53.8%) for an overall rate of 27.5 events per 100 patient-years. Table 2 shows SAE (occurring in at least 1% of patients). Joint surgery related to RA was the most common SAE and occurred in 20 patients (14.0%). In addition, a variety of musculoskeletal disorders was reported as SAE, which were classified as not related to tocilizumab.

**Table 2:** Serious adverse events observed in at least 1% of patients

SAE	No (%)
Any SAE	77 (53.8)
Joint surgery	20 (14.0)
Pneumonia	9 (6.3)
Herpes zoster	7 (4.9)
Tendon rupture	5 (3.5)
Humerus fracture	4 (2.8)
Spinal osteoarthritis	3 (2.1)
Femoral neck fracture	3 (2.1)
Joint dislocation	2 (1.4)
Back pain	2 (1.4)
Lumbar spinal stenosis	2 (1.4)
Bronchitis acute	2 (1.4)
Pyelonephritis	2 (1.4)
Brain stem infarction	2 (1.4)
Cataract	2 (1.4)
Pneumothorax	2 (1.4)
Liver function abnormality	2 (1.4)

SAE, serious adverse event.

Serious infections were reported in 25 patients (17.5%) at a rate of 5.7 events per 100 patient-years. The most frequently reported infections were as follows: pneumonia (nine patients, 1.5 events per 100 patient-years); herpes zoster (seven patients, 1.1 events per 100 patient-years); acute bronchitis (five patients, 0.8 events per 100 patient-years) and pyelonephritis (three patients, 0.5 events per 100 patient-years).

Four malignancies were reported in four patients (2.8%; 0.7 events per 100 patient-years). The types of malignancies were bladder cancer, breast cancer, large intestine carcinoma and intraductal papilloma.

Temporary prolongation of treatment intervals with tocilizumab was observed throughout the study. Although 163 events of prolonged intervals of 8 weeks or more occurred, the majority of the prolongation of intervals was due to transition from the randomised study to the extension study (median interval of the transition was 10.1 weeks). No particular adverse events were reported when tocilizumab was re-administered except for one patient with a severe infusion reaction. The patient had received 4 mg/kg tocilizumab in the initial 3-month trial, and IgE anti-tocilizumab antibodies appeared at the second infusion of the extension trial. Two more patients were positive for anti-tocilizumab antibodies, when tocilizumab was not detectable in their blood. No adverse event was reported related to the anti-tocilizumab antibodies.

**Figure 2:**
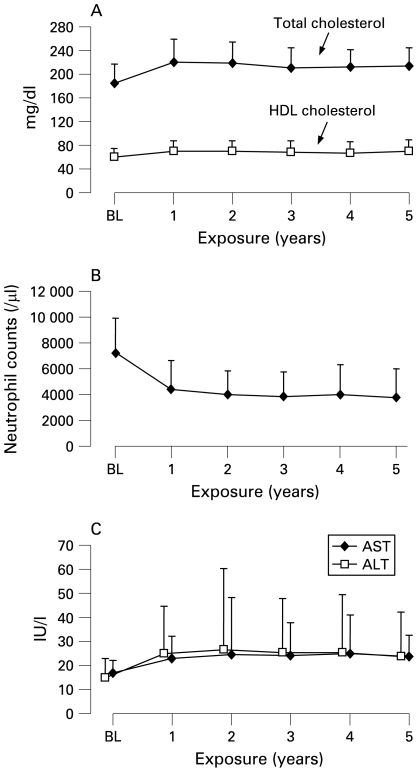
Change in serum total cholesterol, high-density lipoprotein (HDL) cholesterol, neutrophil counts, aspartate aminotransferase (AST) and alanine aminotransferase (ALT). Values are means. Bars indicate SD. BL, baseline.

Mean non-fasting total blood cholesterol increased after treatment initiation and stabilised (mean values 185 mg/dl at baseline; 220 mg/dl at 12 months; 214 mg/dl at 60 months; fig 2A). A total of 112 patients experienced total cholesterol abnormalities at at least one point and 15 patients had abnormal values at baseline. Thirty-nine patients (34.8%) were treated with statins, including two patients who had started statin treatment before the trial. There were no cardiovascular SAE related to tocilizumab except for ischaemic heart disease reported in one patient whose total blood cholesterol increased from 168 mg/dl at baseline to 227 mg/dl without statin treatment. The patient also had the risk factor of diabetes mellitus.

Mean neutrophil counts decreased but remained within the normal range (fig 2B). Grade 2 neutropenia was observed in 17 patients and grade 3 in nine patients. All the events were transient, and no patients experienced febrile neutropenia or withdrew as a result of neutropenia.

Mean aspartate aminotransferase (AST) and alanine aminotransferase (ALT) increased slightly, but remained roughly within the normal ranges (fig 2C). Grade 2 or higher increases in AST and ALT occurred in nine (6.3%) and 14 (9.8%) of 143 patients, respectively, during the study, but most were transient and resolved without any particular treatment. No serious liver disorders, such as fulminant hepatitis, were seen during this study.

### Efficacy

**Figure 3:**
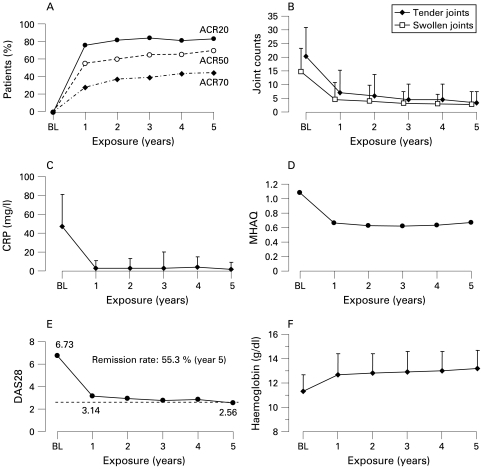
Percentage of responders according to the American College of Rheumatology improvement criteria and the disease activity score in 28 joints (DAS28) as well as the mean change in modified health assessment questionnaire (MHAQ) scores, number of tender joints, number of swollen joints, C-reactive protein (CRP) and haemoglobin. BL, baseline.

The response rate according to the ACR improvement criteria increased during the initial year and remained constant throughout the study period (fig 3A). At 5 years, 79 (84.0%), 65 (69.1%) and 41 (43.6%) of 94 patients met ACR20, ACR50 and ACR70, respectively. These response rates analysed with the last observation carried forward were 77.3%, 58.9% and 37.6%, respectively.

Tocilizumab treatment significantly improved all measures, including tender joint counts, swollen joint counts (fig 3B), CRP levels (fig 3C), MHAQ score (fig 3D) and DAS28 score (fig 3E), and the efficacy was sustained throughout the 5-year treatment. The percentage of patients who achieved clinical remission defined as DAS28 less than 2.6^13^ ^14^ was 55.3% (52/94) at 5 years. Most patients exhibited anaemia at baseline and the mean haemoglobin level was 11.3 mg/dl (SD 1.4). Tocilizumab treatment significantly improved anaemia in these patients, and the mean haemoglobin level was increased to 13.2 mg/dl (SD 1.5) at year 5 (fig 3F).

Eighty-eight of the 94 patients who received tocilizumab for more than 5 years had received corticosteroids when they began the initial study. After 5 years of tocilizumab treatment, 78 of 88 (88.6%) had been able to decrease their corticosteroid dose and 28 of 88 (31.8%) had discontinued corticosteroids. The mean dose of corticosteroids for these patients decreased from 6.9 mg/day (median 7.5 mg/day) to 2.4 mg/day (median 2.0 mg/day) at 5 years.

## Discussion

The STREAM study is the first study demonstrating the long-term safety and efficacy of tocilizumab monotherapy. This open-label extension trial of tocilizumab demonstrated a sustained good efficacy and a generally good safety profile over 5 years. The high retention rate at 5 years indeed indicates the favourable efficacy and safety profile. In particular, only one of 143 patients withdrew as a result of an unsatisfactory response, indicating that no general loss of response occurred during long-term treatment.

ACR responses and improvements in DAS28 scores and individual components of the ACR core set were all sustained during the long-term treatment with tocilizumab monotherapy. At 5 years, approximately half of patients had achieved ACR70 and more than half of patients had achieved clinical remission defined as a DAS28 of less than 2.6, although this study was open labelled.

Tocilizumab monotherapy markedly improved inflammation markers such as CRP and ESR and improvements were sustained throughout the study. Haemoglobin levels were also improved. It is well documented that hepcidin plays a key role in anaemia of chronic inflammatory diseases. IL-6 induces the secretion of hepcidin, an iron regulatory peptide hormone that is produced in the liver and that negatively regulates the absorption of intestinal iron and iron recycling by macrophages.^15^ This increase in haemoglobin levels is expected to contribute to the improvement in patients’ quality of life.

A steroid-sparing effect was another benefit of tocilizumab therapy for RA patients. As the use of corticosteroids is often associated with adverse events such as infection or steroid-induced osteoporosis, this also contributes to the improvement in patients’ quality of life from the safety point of view.

A major objective of this study was to evaluate long-term safety. Long-term treatment with tocilizumab was well tolerated. Most of the adverse events were mild and acceptable compared with the benefit provided. The rate of serious infections of 5.7/100 patient-years after 612 patient-years of treatment was comparable to that reported with tumour necrosis factor (TNF) antagonists.^16^ ^17^ There was no systemic opportunistic infection or tuberculosis in this study. At least two patients with a history of tuberculosis were treated with tocilizumab because this study did not exclude patients who had a history of tuberculosis. Neither had any recurrence nor exacerbation of tuberculosis without the prophylactic use of antituberculosis drugs. However, two cases of tuberculosis were reported in another study (two cases in 1891 patient-years in Japan),^18^ and we should therefore follow patients carefully during tocilizumab treatment.

Four malignancies were reported in four patients. Yamanaka *et al*^19^ reported a comparison of the incidence of malignancies in the following three populations: (1) tocilizumab cohort: all clinical trials (including this trial) of tocilizumab in active RA patients; (2) IORRA cohort: an observational cohort of RA patients in the Institute of Rheumatology, Tokyo Women’s Medical University and (3) a Japanese population database: cancer incidence in Japan by the research group for population-based cancer registration in Japan supported by the Japanese Ministry of Health, Labour and Welfare. The incidence of malignancies in the patients receiving tocilizumab was almost equivalent to that in the observational cohort of RA patients or the Japanese population data. Further study will be required to evaluate whether tocilizumab treatment might influence the incidence of malignancies using a much larger population of RA patients treated with tocilizumab.

Throughout long-term treatment, a serious infusion reaction was observed in only one patient who received 4 mg/kg tocilizumab in the initial double-blind trial and developed IgE anti-tocilizumab antibodies. Maini *et al*^6^ reported that anaphylaxis and anaphylactoid reactions occurred only at low doses of tocilizumab in the absence of methotrexate. Therefore, initial treatment with a relatively low dose (4 mg/kg) of tocilizumab without methotrexate may induce anti-tocilizumab antibodies.

Increases in total cholesterol, high-density lipoprotein cholesterol and triglycerides were observed in the initial controlled study. In this extension study, however, they did not continue increasing. Furthermore, the atherogenic index, calculated by (total cholesterol–high-density lipoprotein cholesterol)/high-density lipoprotein cholesterol, was stable throughout the 5-year treatment. Therefore, an increase in total cholesterol does not always mean an increased risk of cardiovascular disease. As IL-6 is thought to play a causative role in atherosclerosis, IL-6 blockade may decrease the incidence of cardiovascular events, as observed with anti-TNF therapy.^20^ Further investigation will be required to evaluate whether tocilizumab might increase the risk of developing ischaemic heart disease. At present, we should introduce treatment according to the guideline for cholesterol management.

Neutropenia was also reported, as seen in previous studies,^4^ ^5^ ^6^ ^7^ ^9^ but the incidence was less frequent than that observed in combination with methotrexate therapy.^6^ ^7^ ^9^ This may be an advantage of tocilizumab monotherapy.

Although it has been established that TNF inhibitors should be given with methotrexate for maximal efficacy,^21^ ^22^ this study indicated that tocilizumab monotherapy offered a good safety profile and sustained efficacy throughout long-term treatment. Therefore, tocilizumab has considerable clinical benefit for patients who do not tolerate methotrexate. Short-term safety and efficacy studies of tocilizumab in combination with methotrexate or DMARD have been reported,^6^ ^7^ ^8^ ^9^ but further studies are required to determine long-term safety and efficacy.

In conclusion, this study clearly demonstrates excellent long-term efficacy and generally good safety of tocilizumab monotherapy in active RA patients.
